# A human model of inflammatory cardio-metabolic dysfunction; a double blind placebo-controlled crossover trial

**DOI:** 10.1186/1479-5876-10-124

**Published:** 2012-06-18

**Authors:** Nehal N Mehta, Sean P Heffron, Parth N Patel, Jane Ferguson, Rachana D Shah, Christine C Hinkle, Parasuram Krishnamoorthy, Rhia Shah, Jennifer Tabita-Martinez, Karen Terembula, Stephen R Master, Michael R Rickels, Muredach P Reilly

**Affiliations:** 1 Cardiovascular Institute, Perelman School of Medicine at the University of Pennsylvania, Philadelphia, PA, USA; 2 Institute for Diabetes, Endocrine and Metabolism, Perelman School of Medicine at the University of Pennsylvania, Philadelphia, PA, USA; 3 Department of Pathology and Laboratory Medicine, Perelman School of Medicine at the University of Pennsylvania, Philadelphia, PA, USA; 4 Cardiovascular Institute, Perelman School of Medicine at the University of Pennsylvania, 6 Penn Tower, 3400 Civic Center Blvd, Philadelphia, PA, USA

**Keywords:** Inflammation, Obesity, Atherosclerosis, Insulin resistance

## Abstract

**Background:**

Chronic inflammation may contribute to insulin resistance (IR), metabolic syndrome and atherosclerosis although evidence of causality is lacking in humans. We hypothesized that very low-dose experimental endotoxemia would induce adipose tissue inflammation and systemic IR during a low-grade but asymptomatic inflammatory response and thus provide an experimental model for future tests of pharmacologic and genomic modulation of cardio-metabolic traits in humans.

**Methods:**

Ten healthy, human volunteers (50% male, 90% Caucasian, mean age 22.7 ± 3.8) were randomized in a double-masked, placebo-controlled, crossover study to separate 36-hour inpatient visits (placebo versus intravenous-LPS 0.6 ng/kg). We measured clinical symptoms via the McGill pain questionnaire and serial vital signs. Plasma and serum were collected for measurement of cytokines, C-reactive protein, insulin and glucose, serial whole blood & subcutaneous adipose tissue mRNA expression were measured by real-time PCR. HOMA-IR, a well-validated measure of IR was calculated to estimate insulin resistance, and frequently sampled intravenous glucose tolerance testing (FSIGTT) was performed to confirm an insulin resistant state. We performed ANOVA and within subject ANOVA to understand the differences in cytokines, adipose tissue inflammation and IR before and after LPS or placebo.

**Results:**

There was no significant difference between placebo and LPS in clinical responses of symptom scores, body temperature or heart rate. However, low-dose endotoxemia induced a rapid and transient 25-fold induction of plasma TNF-alpha and 100-fold increase in plasma IL-6 (Figure 1B) (*p* < 0.001 for both) both peaking at two hours, followed by modest inflammation in adipose tissue with increases in mRNA levels of several inflammatory genes known to modulate adipose and systemic insulin resistance. Adipose tissue mRNA levels of IL-6 (peak 6-fold, ANOVA F = 27.5, *p* < 0.001) and TNF-alpha (peak 1.8-fold, F = 2.9, *p* = 0.01) increased with MCP-1 (peak 10-fold, F = 5.6, *p* < 0.01) and fractalkine (CX3CL1) (peak 15-fold, F = 13.3, *p* < 0.001). Finally, HOMA-IR was 32% higher following LPS compared to placebo (*p* < 0.01) and insulin sensitivity declined by 21% following LPS compared to placebo (*p* < 0.05).

**Conclusions:**

We present a low dose human endotoxemia model of inflammation which induces adipose tissue inflammation and systemic insulin resistance in the absence of overt clinical response. Such a model has the potential for broad and safe application in the study of novel therapeutics and genomic influences in cardio-metabolic disease.

## Background

Chronic inflammation is a central feature in the pathophysiology of insulin resistance (IR) and diabetes [1-3], both of which are risk factors for the development of atherosclerosis [4]. Indeed, IR and overt type-2 diabetes may emerge during human infections and sepsis [5] via activation of toll-like receptor 4 (TLR-4) signaling. TLR4 also may be activated by endogenous ligands that are increased in diet induced obesity and IR [6]. Further, experimental studies in mouse models of TLR4 deficiency demonstrate a reduction in diet induced obesity [6] and atherosclerosis [7].

The TLR4 pathway can be activated in humans by exogenous administration of standardized preparations of lipopolysaccharide (LPS) under Food and Drug Administration oversight. We and others have shown that insulin resistance [8,9], adipose tissue inflammation [10] and atherogenic lipoprotein changes [11,12] observed acutely during this experimental stimulation resemble those observed chronically in obesity, insulin resistance and atherosclerosis. However, our prior experimental work, which used a moderate dose of LPS (3 ng/kg) [8], was associated with clinical symptoms and modulation of counter-regulatory hormones which limit the potential broad application for the study of cardiometabolic diseases.

There is a great need for development of a feasible human model of low grade inflammation in which novel therapies and endogenous exposures, such as genotypic variation, can be tested for their modulation of inflammatory atherogenic stress. In this paper, we address the hypothesis that low dose endotoxemia (LPS 0.6 ng/kg) induces biologically relevant inflammatory metabolic changes in the absence of overt clinical responses. Our goal was to investigate the feasibility of this potential subclinical model in humans in order provide a tool for testing drugs, diets and genes that modulate inflammatory cardio-metabolic disease.

## Methods

### Endotoxemia clinical protocol

We performed a double blind, placebo-controlled, random sequence, crossover trial of young, healthy, non-smoking males and females (n = 10) who had no vascular disease, diabetes, kidney or liver dysfunction, active infection, elevated glucose, dyslipidemia, hypertension, nor treatment with anti-hypertensive or lipid-modifying medications. After informed consent, participants met with a Clinical Translational Research Center (CTRC) dietician for a weight maintenance diet (controlled for saturated fat and cholesterol intake) to start two-weeks before each of the two 36- hour stays. They were admitted to the CTRC at 6 pm of Day 0, given a standard meal at 7 pm and administered a McGill Short Form Pain Questionnaire [13] and an intravenous (IV) catheter was placed for the administration of saline (100 mL/h) at 10 pm. At 5:30 am on Day 1, another IV catheter was placed for the purpose of serial blood sampling, and baseline blood was drawn prior to slow administration, over three minutes, of lipopolysaccharide (LPS) (0.6 ng/kg, US standard reference endotoxin; lot# CC-RE-LOT-1 + 2 from Clinical Center, National Institutes of Health) or placebo (saline). Following injection, a standardized breakfast was provided.

Serial blood samples and the McGill Short Form Pain Questionnaire were taken at one, two, four, six, 12, 18 and 24 hours following the LPS or placebo intervention. Blood pressure was measured every 15 minutes for the first eight hours following LPS/placebo administration and hourly for the remaining 16 hours of the stay. Heart rate was measured hourly for the first eight hours post-intervention and then at 12, 16 and 24 hours. Subcutaneous adipose tissue biopsies under local anesthesia (1% lidocaine) were obtained 5 minutes prior to LPS/placebo injection and at four, 12 and 24 hours following injection. The four serial subcutaneous gluteal adipose biopsies were performed from a different site each time - i.e. left upper, left lower, right upper, and right lower regions. Subjects were also provided standardized meals at 12 pm and 6 pm on Day 1. Twenty-four hours following the administration of LPS or placebo (approximately 6:00 am on Day 2), a frequently sampled intravenous glucose tolerance test (FSIGTT) was performed to calculate insulin sensitivity [14]. The crossover CTRC visit occurred one month following the first visit in which identical procedures and protocol were followed.

### Blood measures

Serial blood draws occurred in tubes containing EDTA, placed on ice briefly and centrifuged for the isolation of plasma, which was divided into aliquots and stored at −80°C until the time of analyses. Plasma levels of insulin, growth hormone and cortisol (radio-immunoassays (RIA), Linco Research, St Charles, MO), and as previously described [8,10,15], plasma levels of tumor necrosis factor alpha (TNF), interleukin-6 (IL-6) (Linco Multiplex ELISAs on Luminex IS100; Austin TX) were measured in duplicate per manufacturers’ guidelines. Plasma lipids and glucose were measured enzymatically (Wako Diagnostics, Richmond, VA) on a Hitachi 912 automated chemistry system in a Center for Disease Control-certified lipid laboratory as described previously [15,16], and biomarker C-reactive protein (CRP; high sensitivity) levels were assayed using immunoturbidemetry as described [17].

### Adipose tissue biopsy and RNA expression

Adipose tissue samples obtained via needle aspiration biopsy as described [8,10,15] were treated with RNA Later® (Qiagen, Valencia, CA), snap frozen and stored at −80°C. Fat tissue RNA was extracted with the RNeasy total RNA kit (Qiagen, Valencia, CA), providing approximately 1–4 g RNA per 100 mg tissue, and subjected to RT-PCR followed by quantitative PCR (qPCR) on an Applied Biosystems 7300 Real-Time PCR System (ABI, Foster City, CA). Adipose tissue mRNA levels of IL-6, TNF, suppressor of cytokine signaling (SOCS)-1, SOCS-2, SOCS-3, SOCS-6, monocyte chemoattractant protein (MCP)-1 and fractalkine (CXC3L1) were determined as we have described [8,10,15]. Variability in total cDNA concentrations between samples was normalized by subtracting the beta-actin C_t_ value from the target C_t_ value for each sample. The comparative C_t_ method was used to analyze changes in gene expression [18]. The ∆C_t_ for each post-LPS sample was compared to the mean ∆C_t_ for all pre-LPS samples in a single individual using the relative quantitation 2^-(∆∆Ct)^ method to determine fold-change from baseline.

### Insulin sensitivity

#### *Estimation of insulin sensitivity and pancreatic beta-cell function*

Twenty four hours prior to LPS and 24 hours following LPS, the insulin sensitivity index (SI) was derived from the frequently sampled intravenous glucose tolerance test (FSIGTT). We chose the FSIGTT as the method for determining insulin sensitivity since the test also provides a measure of pancreatic beta-cell function via the acute insulin response to glucose (AIRg), allowing simultaneous assessment for an effect of endotoxemia on the beta-cell. The FSIGTT was conducted using the insulin-modified approach as previously described [19]. SI was derived from Bergman’s minimal model [14,20] using MINMOD Millennium software [21]. Of the 10 subjects (20 potential FSIGTT exams), we had complete data at both visits in five subjects; two subjects had inconsistent glucose administrations at one of the visits, two subjects had inconsistent timing of insulin administration at one of the visits and one subject had extreme outlier data (>2.5 standard deviations for insulin and glucose). Complementary estimates of insulin resistance and beta-cell function, the homeostasis model assessment for insulin resistance, HOMA-IR index [glucose (mmol/L) x insulin (μU/mL)/22.5], and the HOMA for beta-cell function, HOMA-B index [insulin (μU/mL) x 20/glucose (mmol/L) - 3.5], were calculated independently of FSIGTT data using fasting glucose and insulin values at 24 hours after placebo and 24 hours after LPS.

### Statistical analyses

Data are presented as mean and standard deviation for continuous variables and frequencies for categorical variables. Baseline characteristics between males and females were tested using t- tests for normal data and Kruskal-Wallis testing for non-parametric data. The effect of LPS on clinical parameters, plasma biomarkers, metabolic measures and adipose tissue mRNA levels were tested by repeated measures analysis of variance (ANOVA). When significant global differences were found in ANOVA, post hoc paired t-tests were used to compare time-points. Data are presented in the figures as mean and standard error of the mean. Analyses were performed using STATA 12.0 (College Station, TX). Statistical significance was defined as a *p*- value <0.05. We did not correct for multiple testing; plasma cytokine data represent our primary endpoints with additional traits analyzed to provide complementary information regarding the model impact on diverse cardio-metabolic and biological pathways.

## Results

### Baseline characteristics of participants

Participants were healthy volunteers (50% male, 90% Caucasian, age 22.7 ± 3.8) with normal blood pressure, plasma lipoproteins and BMI as well as expected gender differences in HDL-C, TG and CRP levels (Table 1). The baseline glucose, SI and HOMA-IR measurements were consistent with an insulin sensitive, glucose-tolerant healthy sample.

**Table 1:** **Baseline characteristics of Study Participants (N =** **10)**

		**Mean (SD)**
**Age (years)**		22.7 (3.8)
	**Males**	20.4 (3.2)
	**Females**	25.0 (2.9)
**BMI (kg/m**^**2**^**)**		24.0 (2.3)
**Waist Circumference (m)**		0.85 (0.06)
**TNF-α (pg/mL)**		0.9 (0.4)
**IL-6 (pg/mL)**		1.5 (0.8)
**CRP (mg/L)**		1.2 (1.4)
	**Males**	0.5 (0.2)
	**Females**	1.8 (1.7)
**Systolic Blood Pressure (mmHg)**		115.5 (7.2)
**Diastolic Blood Pressure (mmHg)**		69.1 (3.3)
**Heart rate (bpm)**		58.9 (9.9)
**Temperature (K)**		309.3 (255.4)
**Total Cholesterol (mmol/L)**		4.42 (0.83)
**HDL-C (mmol/L)**		1.54 (0.33)
	**Males**	1.25 (0.09)
	**Females**	1.83 (0.19)
**Triglycerides (mmol/L)**		0.89 (0.34)
	**Males**	0.76 (0.25)
	**Females**	1.03 (0.39)
**LDL-C (mmol/L)**		2.22 (0.56)
*FSIGTT-insulin sensitivity index (SI, x 10^**-4**^**(μU/ml)**^**-1**^**·min**^**-1**^**)**		3.4 (1.1) ^
†**HOMA-IR insulin resistance index**		10.4 (2.77)
**(AIRg, μU·ml**^**-1**^**·min)**		400.5 (152.6) ^
†**HOMA-B beta-cell function index**		1555.6 (931.3)

*FSIGTT = Frequently Sampled Intravenous Glucose Tolerance Test. † HOMA = Homeostatic Model Assessment ^ In N = 5 subjects.

### Systemic inflammatory response to low-dose endotoxemia

**Figure 1:**
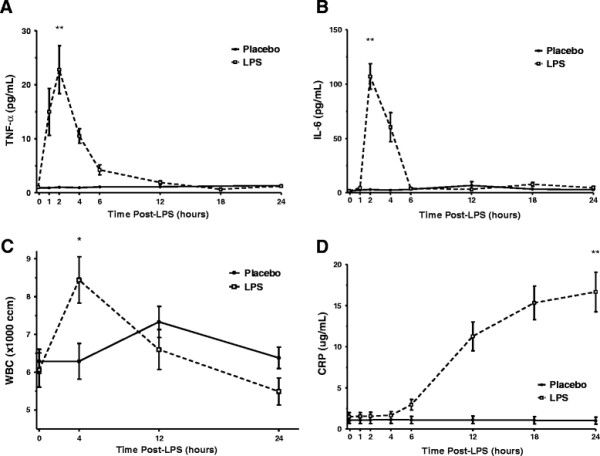
Endotoxemia led to rapid increases in (A) TNF-α (**p < 0.001 vs. placebo, peak 2 hours) and (B) IL-6 (**p < 0.001 vs. placebo, peak 2 hours). Endotoxemia also increased (**C**) white blood cell counts (*p < 0.05 vs. placebo, peak 4 hours) and (**D**) CRP levels (*p < 0.001 vs. placebo, peak 24 hours). Data presented as mean ± SEM (n = 10).

Low-dose endotoxemia induced a rapid and transient induction of plasma TNF-alpha (Figure 1A) and IL-6 (Figure 1B) (*p* < 0.001 for both) both peaking at two hours. There was a later increase in white blood cells (Figure 1C) (peak four hours, *p* = 0.007), and subsequent increase of the biomarker, CRP (Figure 1D) (highest level during twenty four hour assay period at 24 h post LPS, *p* < 0.001). These findings confirm the expected transient but robust cytokine and biomarker response to low-dose endotoxemia.

### Clinical and counter-regulatory responses to low-dose endotoxemia

**Figure 2:**
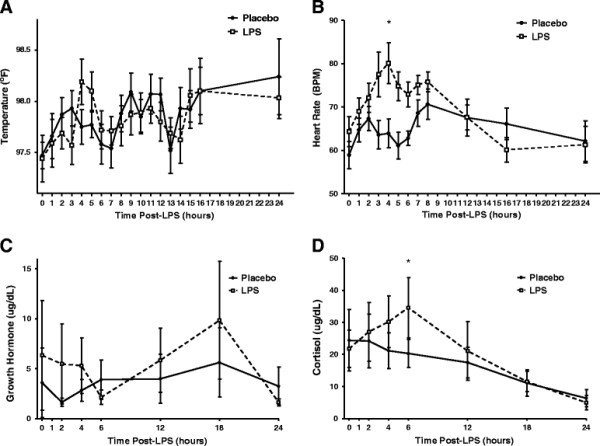
Endotoxemia produced no difference in (A) temperature, though (B) heart rate increased modestly from 4–8 hours relative to placebo (*p < 0.05, peak 4 hours). In addition, endotoxemia had no significant modulation in (**C**) growth hormone (*p *= 0.71, peak 18 hours), but had a transient increase in (**D**) serum cortisol (*p < 0.05, peak 6 hours). Data presented as mean ± SEM in panels **A**, **B**, and 95% CI in panels **C**, **D **(n = 10).

Following low dose endotoxin or placebo administration, there was no significant difference in subjective pain and clinical symptoms as assessed by the McGill questionnaire Visual Analogue Scale (VAS) (within subject ANOVA following LPS, *p* = 0.2) and Present Pain Intensity (PPI) (within subject ANOVA following LPS, *p* = 0.12). The slight trend in scores did not reach a statistically significant difference between LPS and placebo and is unlikely to be of clinical significance when placed in context of the typical flu-like symptomatic response to moderate dose LPS (3 ng/kg) (Additional file 1: Figure S1). In addition, we observed no significant differences in body temperature (Figure 2A) or blood pressure (not shown) following low dose LPS compared to placebo while heart rate (Figure 2B) increased modestly at the 4–8 hour timeperiod following LPS (*p* = 0.04). There was a trend toward small increases in growth hormone (Figure 2C) (peak trend at 18 hours, *p* = 0.71) and serum cortisol (Figure 2D) (peak change at six hours, *p* < 0.05) following LPS. These data suggest a subclinical inflammatory response with very modest counter-regulatory hormone activation.

### Low-dose endotoxemia induces adipose tissue inflammation

**Figure 3:**
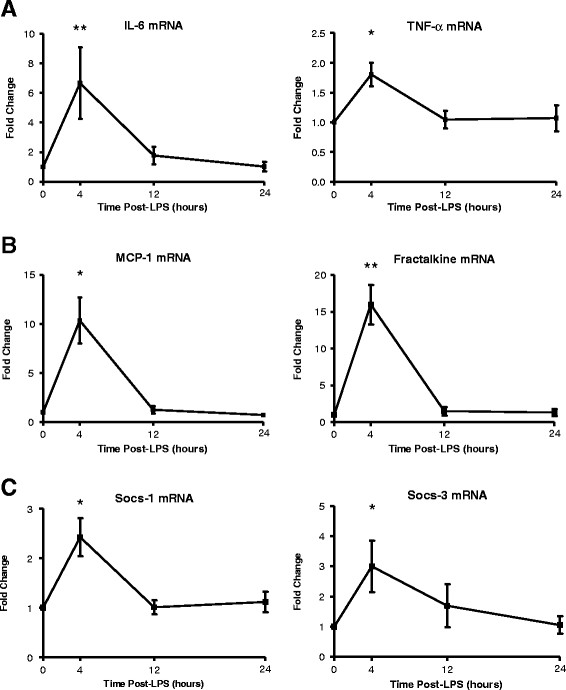
**Endotoxemia increased adipose mRNA levels of cytokines (A) IL-6 and TNF-α, chemokines (B) MCP-1 and fractalkine and (C) SOCS-1 and SOCS-3. **(*p  < 0.05, **p < 0.001). Data presented as mean ± SEM (n = 10).

Low-dose endotoxemia induced modest inflammation in adipose tissue with increase in mRNA levels of several inflammatory genes known to modulate adipose and systemic insulin resistance (Figure 3). Thus, adipose tissue mRNA levels of IL-6 (peak 6-fold, ANOVA F = 27.5, *p* < 0.001) and TNF-alpha (peak 1.8-fold, F = 2.9, *p* = 0.01) increased with MCP-1 (peak 10-fold, F = 5.6, *p* < 0.01) and fractalkine (CX3CL1) (peak 15-fold, F = 13.3, *p* < 0.001), chemokines involved in monocyte and T-cell recruitment and implicated in adipose tissue inflammation and insulin resistance [22,23]. Previously, we have shown increases in levels of MCP1 [8] and fractalkine [10] proteins in adipose tissue following higher doses of LPS. Members of the suppressor of cytokine signaling (SOCS) family of proteins inhibit tyrosine kinase receptor signaling and are known to attenuate the insulin receptor and induce adipose insulin resistance [24,25]. Two of these, SOCS-1 (2.5-fold, *p* = 0.01) and SOCS-3 (3-fold, *p* < 0.01) mRNAs increased modestly following low dose LPS. We did not observe significant changes in anti- inflammatory cytokine IL-10 or in SOCS 2 and SOCS 6 following LPS (data not shown). 

### Low-dose endotoxemia induces systemic insulin resistance in humans

**Figure 4:**
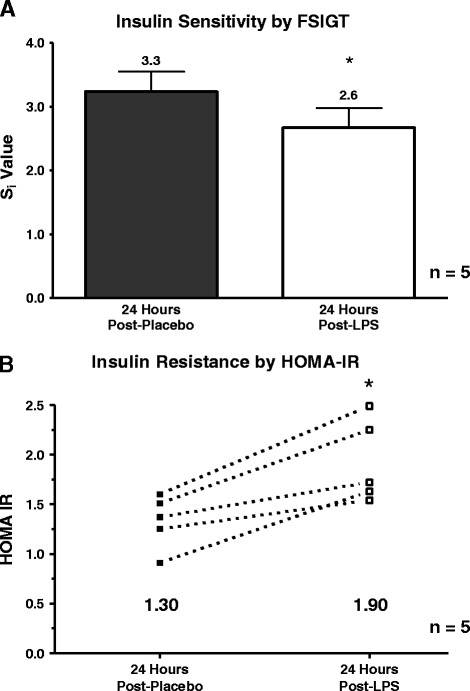
**Endotoxemia led to a systemic insulin resistant state as shown by (A) a decrease in the insulin sensitivity index (S**_**i**_**) at FSIGT testing (placebo 3.31 ± 0.62 vs LPS 2.60 ± 0.63 (μU/ml)**^**- 1**^**·min**^**-1**^, *p < 0.05, n = 5. Consistent with this decrease in insulin sensitivity, endotoxemia increased (**B**) HOMA-IR compared to placebo (*p < 0.05, n = 5).

We previously demonstrated that higher dose (3 ng/Kg) endotoxemia induced acute systemic IR without altering pancreatic beta-cell function [8]. However, at that dose the clinical and inflammatory changes were much greater than that observed in obesity and metabolic syndrome. Here, we observed a more modest decrease in insulin sensitivity at FSIGTT (n = 5) following low-dose endotoxemia; insulin sensitivity (SI) declined by 21% following LPS compared to placebo (*p* < 0.05) (Figure 4A) with no significant change in the AIRG index of pancreatic beta-cell function (placebo 463.02 ± 161.4 vs. LPS 405.45 ± 157.7 (μU·ml^-1^·min), *p* = 0.58). Consistent with the FSIGT data, insulin resistance estimated by HOMA-IR in the FSIGTT sub-sample (n = 5) was 32% higher following LPS compared to placebo (*p* < 0.01) (Figure 4B) while HOMA-B data, a surrogate of fasting pancreatic beta-cell function, was unchanged (placebo 231.4 ± 140.1 vs. LPS 230.2 ± 84.0, *p* = 0.98). In the entire sample, HOMA IR showed the same pattern of increase (placebo 1.49 ± 0.21 vs. LPS 1.77 ± 0.24, *p* = 0.02) as that observed in the FSIGTT sub-sample with no change in HOMA-B (placebo 235.4 ± 131.4 vs. LPS 232.2 ± 96.1, *p* = 0.9). Overall, low dose endotoxemia produced systemic insulin resistance following induction of specific adipose inflammatory pathways (cytokines, chemokines and SOCS) that attenuate insulin signaling *in vivo*. 

## Discussion

In this double blind, placebo-controlled, random sequence crossover study of low-dose endotoxemia designed to minimize clinical symptoms, we observed several findings which support its potential use in the study of cardiometabolic diseases: 1) a robust biochemical systemic inflammatory response; 2) an almost complete lack of clinical responses; 3) minimal counter regulatory response in cortisol and growth hormone; 4) significant modulation of cytokine, chemokine in adipose tissue and insulin signaling pathways; and 5) induction of systemic insulin resistance without evidence of pancreatic beta cell dysfunction.

Experimental endotoxemia, which stimulates toll-like receptor 4 (TLR-4) signaling *in vivo,* may be an informative model to study cardio-metabolic traits in humans [9,26]. Observational data show that sepsis and chronic infection [27,28] induce insulin resistance, glucose intolerance and lipid derangement resembling that observed in obesity, type-2 diabetes and atherosclerosis. In addition, we and others have shown that experimental endotoxemia induces insulin resistance [8,9], adipose tissue inflammation [10] and atherogenic lipoprotein changes, including impaired reverse cholesterol transport [11,12,26,29,30]. In humans, endogenous TLR4 antigens, including fatty acids [6] and oxidized lipids [31], are generated in obese adipose and atherosclerosis and may drive inflammatory cardio-metabolic dysfunction. Indeed, TLR4 is directly implicated in diet induced obesity [6] and atherosclerosis through studies in TLR4 deficient mouse models. Thus, endotoxemia has strong biological plausibility and activates relevant pathways known to be perturbed in obesity, diabetes and atherosclerosis.

We previously reported systemic inflammatory changes in adipose tissue with development of a systemic insulin resistance state [8] utilizing a moderate dose of endotoxin (3 ng/kg). Despite its utility in studying inflammatory effects on lipoproteins, metabolic function and adipose tissue in human, this proof-of-principle model is supra-physiologic and induces marked changes in systemic inflammatory markers compared to the current low-dose model and compared to that observed chronically in cardio-metabolic disease states. More importantly, moderate-dose endotoxemia is associated with overt clinical responses, including fever, tachycardia and flu-like aches [8,10,15] which may limit wider application in large-scale human clinical research. In the current study, we demonstrate more subtle changes in inflammatory and metabolic responses with an absence of clinical symptoms during low-dose endotoxemia (LPS 0.6 ng/kg). Our findings support the use of this low-dose model in studying clinically relevant metabolic changes while providing a safe and scalable approach for testing novel therapeutics and genomic influences on cardio-metabolic disorders.

In moderate dose endotoxemia [8], we observed strong induction of subcutaneous adipose TNF and IL-6 as well as MCP-1, which is known to recruit CCR-2 expressing monocytes, increase inflammatory-M1 adipose tissue macrophage (ATM) and promote insulin resistance [22]. In support of this concept, we also observed increased mRNA levels of the macrophage marker, EMR1-F4/80 [32], in adipose tissue. Thus, endotoxemia may promote adipose recruitment of macrophages, a characteristic of adipose tissue in obese, insulin resistant humans^33^. In our current study, we demonstrate that low dose endotoxemia produced a more subtle adipose tissue inflammation. However, the increases in adipose cytokines and chemokines as well as induction of SOCS were similar to those observed in diet and obesity-related insulin resistance [22,32-37]. Further, adipose changes coincided with subsequent emergence of modest insulin resistance which was less than that with higher dose endotoxin but consistent with that in metabolic syndrome [38] and diabetes [19]. Thus, low dose endotoxemia also provides a model of inducible adipose tissue inflammation permitting specific interrogation of factors that modulate this important tissue component of insulin resistance.

Epidemiological studies have demonstrated a consistent relationship between chronic low grade inflammation and states of obesity, insulin resistance, diabetes and atherosclerosis. A challenge in understanding the mechanism of these associations in humans however remains hampered by a lack of a reliable *in vivo* model. Here we show that an evoked inflammatory model to simulate these states can be fruitful in identifying genes and pathways activated in cardio-metabolic disease. We acknowledge that the low dose endotoxemia model does not reproduce the chronic pathophysiology of complex cardio-metabolic diseases. It is, however, associated with minimal clinical response and approximates acutely the inflammatory and metabolic responses of the chronic disease states of interest. Furthermore, low-dose experimental endotoxin induction of toll-like receptor 4 (TLR-4) signaling *in vivo* is one well established model of inflammation-induced metabolic disturbances in humans. Sepsis and chronic infections in humans induce insulin resistance, glucose intolerance and lipoprotein changes similar to the metabolic profile observed in obesity, type-2 diabetes and established coronary artery disease. The insulin resistance, adipose inflammation and lipoprotein changes observed acutely during experimental endotoxemia resemble those observed chronically in cardio-metabolic disease states. Finally, the role of TLR4 (the endotoxin signaling receptor) is further suggested by studies demonstrating reduced diet induced obesity and atherosclerosis in TLR4 deficient mouse models. Indeed, in addition to endotoxin, TLR4 may be activated by endogenous ligands that are increased in diet induced obesity and insulin resistance. Of further relevance to obesity and metabolic disease *in vivo*, rodents raised in germ-free conditions are protected from diet induced obesity [39]. Therefore, our model of induced inflammation from acute bacterial exposure is one of several models of inflammation evoked metabolic disturbance *in vivo* and may not exactly mimic all means of generating subclinical inflammation in obesity, diabetes and cardiovascular disease. However, this model also offers the additional advantage in permitting direct assessment of the directional impact of induced inflammation on metabolic parameters such as insulin resistance. This avoids confounding and reverse causation that are features of observational studies where inflammatory changes may result from risk factors and disease rather than be causal.

## Conclusion

In summary, we present a low dose human endotoxemia model of inflammation which induces adipose tissue inflammation and systemic insulin resistance in the absence of overt clinical response. Such a model has the potential for broad and safe application in the study of novel therapeutics and genomic influences in cardio-metabolic disease.

## Abbreviations

IR, Insulin Resistance; TLR-4, Toll-like receptor 4; LPS, lipopolysaccharide; CTRC, Clinical Translational Research Center; IV, Intravenous; FSIGTT, Frequently sampled intravenous glucose tolerance test; EDTA, Ethylenediaminetrichloroacetic acid; RIA, Radio-immunoassays; TNF, Tumor Necrosis factor α; IL, Interleukin; ELISA, Enzyme linked immuosorbent assay; RT-PCR, Real time Polymerase chain reaction; qPCR, Quantitative PCR; SOCS, Suppressor of cytokine signaling; MCP1, Monocyte chemoattractant protein-1; SI, Sensitivity Index; AIRg, Acute insulin response to glucose; HOMA-IR, Homeostatic model assessment of insulin resistance; HOMA-B, Homeostatic model assessment of beta cells of pancreas; ANOVA, Analysis of Variance; BMI, Body Mass Index; HDL-C, High Density Lipoprotein Cholesterol; TG, Triglycerides.

## Competing interests

The authors declare that they have no competing interests.

## Authors’ contribution

NNM and MPR designed and executed the study and wrote the manuscript. SPH, JEF, RDS, CH, KT, RS collected data and revised the manuscript. PNP, PK and MRR analyzed data and revised the manuscript and JTM collected the data. All authors have read and approved the final manuscript.
